# The impact of high frequency oscillatory ventilation on mortality in paediatric acute respiratory distress syndrome

**DOI:** 10.1186/s13054-020-2741-x

**Published:** 2020-01-31

**Authors:** Judith Ju-Ming Wong, Siqi Liu, Hongxing Dang, Nattachai Anantasit, Phuc Huu Phan, Suwannee Phumeetham, Suyun Qian, Jacqueline Soo May Ong, Chin Seng Gan, Yek Kee Chor, Rujipat Samransamruajkit, Tsee Foong Loh, Mengling Feng, Jan Hau Lee, for the Pediatric Acute & Critical care Medicine Asian Network (PACCMAN)

**Affiliations:** 1 Children’s Intensive Care Unit, Department of Pediatric Subspecialties, KK Women’s and Children’s Hospital, 100 Bukit Timah Road, Singapore, 229899 Singapore; 2 Saw Swee Hock School of Public Health, National University Health System, NUS Graduate School for Integrative Science and Engineering, National University of Singapore, 12 Science Drive 2, Singapore, 117549 Singapore; 3 Pediatric Intensive Care Unit, Children’s Hospital of Chongqing Medical University, 136 Zhongshan 2nd Rd, Yuzhong district, Chongqing, 400041 China; 4 Ramathibodi Hospital, Mahidol University, 270 Rama VI Road, Ratchathewi, Bangkok, 10400 Thailand; 5 National Children’s Hospital, 18/879 La Thành, Láng Thượng, Đống Đa, Hanoi, Vietnam; 6 Faculty of Medicine Siriraj Hospital, Mahidol University, 2 Wanglang Road Bangkoknoi, Bangkok, 10700 Thailand; 7 Beijing Children’s Hospital, Capital Medical University, 56 Nanlishi Rd, Xicheng District, Beijing, 100045 China; 8 Khoo Teck Puat-National University Children’s Medical Institute, National University Hospital, 5 Lower Kent Ridge Road, Singapore, 119074 Singapore; 9 Department of Pediatrics, University of Malaya. Jalan Universiti, 50603, Wilayah Persekutuan, Kuala Lumpur, Malaysia; 10 Sarawak General Hospital, Jalan Hospital, 93586 Kuching, Sarawak Malaysia; 11 Critical Care Excellence Center, King Chulalongkorn Memorial Hospital, Faculty of Medicine, Chulalongkorn University Bangkok, Bangkok, 10330 Thailand

**Keywords:** High-frequency ventilation, Mechanical ventilation, Acute respiratory distress syndrome, Acute lung injury, Paediatric intensive care unit, Children

## Abstract

**Background:**

High-frequency oscillatory ventilation (HFOV) use was associated with greater mortality in adult acute respiratory distress syndrome (ARDS). Nevertheless, HFOV is still frequently used as rescue therapy in paediatric acute respiratory distress syndrome (PARDS). In view of the limited evidence for HFOV in PARDS and evidence demonstrating harm in adult patients with ARDS, we hypothesized that HFOV use compared to other modes of mechanical ventilation is associated with increased mortality in PARDS.

**Methods:**

Patients with PARDS from 10 paediatric intensive care units across Asia from 2009 to 2015 were identified. Data on epidemiology and clinical outcomes were collected. Patients on HFOV were compared to patients on other modes of ventilation. The primary outcome was 28-day mortality and secondary outcomes were 28-day ventilator- (VFD) and intensive care unit- (IFD) free days. Genetic matching (GM) method was used to analyse the association between HFOV treatment with the primary outcome. Additionally, we performed a sensitivity analysis, including propensity score (PS) matching, inverse probability of treatment weighting (IPTW) and marginal structural modelling (MSM) to estimate the treatment effect.

**Results:**

A total of 328 patients were included. In the first 7 days of PARDS, 122/328 (37.2%) patients were supported with HFOV. There were significant differences in baseline oxygenation index (OI) between the HFOV and non-HFOV groups (18.8 [12.0, 30.2] vs. 7.7 [5.1, 13.1] respectively; *p* < 0.001). A total of 118 pairs were matched in the GM method which found a significant association between HFOV with 28-day mortality in PARDS [odds ratio 2.3, 95% confidence interval (CI) 1.3, 4.4, *p* value 0.01]. VFD was indifferent between the HFOV and non-HFOV group [mean difference − 1.3 (95%CI − 3.4, 0.9); *p* = 0.29] but IFD was significantly lower in the HFOV group [− 2.5 (95%CI − 4.9, − 0.5); *p* = 0.03]. From the sensitivity analysis, PS matching, IPTW and MSM all showed consistent direction of HFOV treatment effect in PARDS.

**Conclusion:**

The use of HFOV was associated with increased 28-day mortality in PARDS. This study suggests caution but does not eliminate equivocality and a randomized controlled trial is justified to examine the true association.

**Electronic supplementary material:**

The online version of this article (10.1186/s13054-020-2741-x) contains supplementary material, which is available to authorized users.

## Introduction

High-frequency oscillatory ventilation (HFOV) is an alternative mode of mechanical ventilation (MV) that delivers small tidal volumes with low phasic pressure changes at supraphysiologic frequencies [1]. The non-conventional gas exchange mechanisms are expected to produce less ventilator-induced lung injury, and with initial data showing improvements in short-term oxygenation and ventilation, the use of HFOV in intensive care units became popular [2–5]. However, these physiologic improvements did not translate into clinical benefits in two large randomized controlled trials (RCT) of adult patients with acute respiratory distress syndrome (ARDS). The OSCILLATE trial was stopped prematurely (*n* = 548) due to the findings of higher in-hospital mortality in the HFOV group compared to controls [relative risk of death with HFOV 1.33 (95% confidence interval [CI], 1.09 to 1.64)] [6]. The OSCAR trial (*n* = 795) demonstrated no difference in 30-day mortality [1.03 (95%CI 0.75 to 1.40)] [7]. When these were combined with eight other RCTs in a meta-analysis (*n* = 1850), HFOV use did not lead to a significant difference in-hospital or 30-day mortality compared with conventional MV (CMV) [8]. Instead, HFOV use was associated with greater undesirable side effects including the need for more sedatives and vasoactive drugs [6, 9].

Evidence for the use of HFOV remains weak in paediatric acute respiratory distress syndrome (PARDS). Majority of studies conducted thus far are small [10–17]. Similar to studies performed in other populations, paediatric studies of HFOV showed a benefit in short-term oxygenation without any improvement in clinical outcomes [12, 18, 19]. HFOV use in children with acute respiratory failure was associated with increased mortality, duration of MV and paediatric intensive care unit (PICU) stay compared to those who were not supported with HFOV [20, 21]. However, one limitation of these studies is the inclusion of a heterogenous cohort of children with acute respiratory failure. Other studies that included only children with PARDS were small and not able to meaningfully study the effects of HFOV on clinical outcomes [17–19, 22, 23]. Nevertheless, HFOV is still being used frequently in PARDS [24].

In view of the limited evidence for HFOV in PARDS and evidence demonstrating harm in adult patients with ARDS, we hypothesized that HFOV use compared to other modes of MV is associated with increased mortality in PARDS.

## Materials and methods

This study is reported in accordance with the Strengthening the Reporting of Observational Studies in Epidemiology (STROBE) statement. [25] This is a retrospective study of children with PARDS admitted to 10 multidisciplinary PICUs in the Paediatric Acute and Critical Care Asian Network (PACCMAN) and was approved by all participating hospital’s institutional review boards with waiver of consent.

### Datasets

Identification of patients and data collection methods have been described in detail previously [24]. In brief, patients on invasive MV were identified over the study period 2009–2015 according to the Paediatric Acute Lung Injury Consensus Conference (PALICC) criteria for PARDS [26]. The Research Electronic Data Capture (REDCap) system was used for secure remote multi-site data entry and centralized data management. [27]

The “HFOV group” was defined by any use of HFOV within the first 7 days of PARDS. In general, centres used HFOV as a rescue mode of ventilation when there was oxygenation or ventilation failure despite high ventilatory settings or when air leaks were present. Initiation, optimization and discontinuation were at the discretion of the respective primary PICU physicians. The “non-HFOV group” consisted of patients on all other modes of MV (e.g. pressure control ventilation, volume control ventilation, pressure support, airway pressure release ventilation), whereas CMV referred to pressure- and volume-controlled ventilation only. In general, centres observed lung protective ventilation strategies with tidal volumes aimed at 6–8 ml/kg on CMV and accepted permissive hypercapnia and permissive hypoxia.

The primary outcome was 28-day mortality. Secondary outcomes included 28-day ventilator-free days (VFD) and 28-day intensive care unit-free days (IFD). VFD is defined as days alive and free from MV up to 28 days. If a patient is extubated on day 2 and remain alive during the remaining 28 days without using MV, then his/her VFD is 26; whereas a patient who died within the 28-day period, then the VFD score is 0. IFD is defined as days alive and discharged from the PICU up to 28 days. This is to eliminate mortality as a competing interest in evaluating MV and PICU duration.

### Statistical analysis

Categorical and continuous variables were presented as counts (percentages) and median (interquartile range), respectively. We analysed HFOV treatment effect by matching patients in HFOV and non-HFOV groups using genetic matching (GM) [28, 29]. Covariates were chosen before matching and the choice was based on previous empirical analyses and expert opinion [20, 29, 30]. Potential confounding factors include patient demographics [age, gender, comorbidities, multiple organ dysfunction (MOD)], disease severity scores [paediatric index of mortality 2 (PIM2) score, paediatric logistic organ dysfunction (PELOD) score], presence of bacteraemia, risk factors for PARDS (pneumonia, sepsis, aspiration, transfusion and drowning) and oxygenation index (OI) [31, 32]. We used OI at 24 h after admission to PICU in our main analysis as this was reported to be a better predictor of outcomes compared to initial oxygenation values [31, 33, 34]. Daily OI values during the first week of PICU were also available with imputation. Missing values was imputed by the particular patient’s values before and after the missing data. To avoid introducing bias from imputation, we included all the analysis with daily OI in the supplementary material as confirmation of effect direction rather than the true estimate. To assess the multi-centre effect of HFOV treatment among the 10 centres, we applied Cox proportional hazard (CPH) model stratified by centres.

#### Genetic matching

GM is a method that combines matching on the propensity score (PS) and individual covariates, using the Mahalanobis distance [35]. The GM is non-parametric and does not depend on knowing or estimating the PS, but the method is greatly improved when an estimated PS is incorporated [28]. PS is the conditional probability of having HFOV treatment given the confounding factors. We first estimated the PS by fitting the logistic regression model to both non-HFOV and HFOV groups to estimate their probability of receiving the HFOV at the time of PARDS diagnosis. We applied fivefold cross validation on the PS model to ensure that the model did not overfit. We assessed the performance of the PS model by looking at the area under the receiver operating characteristic curve (AUROC). Subsequently, GM optimized the covariate balance between the matched pairs from HFOV and non-HFOV groups. All the aforementioned confounding factors were included as covariates, and the PS is included as an additional covariate in the GM model. GM selects matched pairs using a generalized Mahalanobis distance metric, which includes a vector of weights that indicates the relative importance for each individual covariate. The higher the weight, the more important the covariate as a confounding factor. Expert opinion was used to designate which of covariates were high- or low-priority variables to balance. For instance, the most important confounder was anticipated to be OI, which was a key indicator when inferring mortality [31, 34]. In GM, the weights could be initialized with prior knowledge, and it was optimized by an automated search algorithm, so that the weights would give the best covariate balance in the matched pairs. Thus, GM automates the process of maximizing balance on observed covariates in the matched subjects. We performed GM with replacement and checked the covariate balance through the standardized difference after matching. The association between HFOV and 28-day mortality was analysed using McNemar’s test, while the secondary outcomes were analysed using the Kruskal-Wallis test. Results of the GM were reported using odds ratios (ORs) and the corresponding 95% confidence interval (CI). All statistical significance was inferred when *p* value < 0.05. The detailed explanation on the algorithm and formula of GM is given in Additional file 1: “SE1: Genetic Matching”.

To test the robustness of GM, we applied subgroup analysis to investigate if there is any difference of the OR for different subgroups. Here, we conducted 10 experiments whereby each experiment was done by dropping one centre’s subjects out and re-matching the pairs using the remaining subjects. This experiment was repeated for all the 10 centres. In addition, we performed another four subgroup analyses with GM for (1) age ≥ 1 year vs. age < 1 year, (2) direct vs. indirect PARDS, (3) severe vs. non-severe PARDS and (4) MOD vs. no MOD. Covariate balance was assessed in each experiment.

#### Sensitivity analysis

We performed sensitivity analysis including PS matching, inverse probability of treatment weighting (IPTW) [36] and marginal structural model (MSM) [37, 38] to confirm our findings on the association of HFOV treatment with outcome [39–41]. PS matching was performed with one-to-one matching using a PS with calliper 0.01 across HFOV and non-HFOV groups. Balance was assessed using standardized difference and *p* values. An extended analysis using *daily* PS matching (i.e. patients were matched on a daily basis across the two groups) was done (Additional file 1: SE2). For IPTW approach, the HFOV group was weighted by 1/PS, and the non-HFOV group was weighted by 1/(1-PS), creating a pseudo-population in which the distributions of the confounding factors among the HFOV and non-HFOV groups are balanced, i.e. making the control and the treatment groups interchangeable [42]. Details for applying IPTW can be found in Additional file 1: SE3. Balance of the weighted cohort was assessed using standardized difference and *p* values. Analyses from the PS matching and IPTW model was reported using OR and corresponding 95% CI. MSM was additionally performed to incorporate the effect of the time-dependent HFOV exposure during the first 7 days of ICU stay and thus obtained stabilized weights [43, 44]. The MSM was constructed by fitting the CPH model with the stabilized weighted cohort to estimate the association between HFOV use and outcome. Validity of the proportional hazard assumption was checked using statistical R package ‘survival’ with function ‘cox.zph’ [45]. Analyses from MSM CPH model was reported using hazard ratio (HR) and corresponding 95% CI for the HFOV treatment and all covariates. In theory, MSM has the advantage of accounting for time-dependent treatment effects and time-dependent confounding factors, and is more likely to produce an unbiased estimate of the treatment effect; it was not used as the primary analysis because the model is complex and requires larger data volume to fit in [46, 47]. More details of the MSM approaches are included in Additional file 1: SE4. In addition, we applied multivariate logistic regression and considered HFOV as a predictor variable for 28-day mortality together with other confounding factors to examine the impact of HFOV on mortality. The full reproducible code is available on Github [48]. The analysis was conducted on R 3.5.0 [49], with the survival [50], Matching [51], ipw [36], survey [52], tableone [53] and optmatch [54] packages.

## Results

A total of 427 patients fulfilled our inclusion criteria for PARDS. In this analysis, 328 PARDS patients had complete data and were included in the analysis (Table 1). Characteristics of the selected cohort were similar to the original cohort (Additional file 1: Table S1). 122/328 (37.2%) patients were supported on HFOV during their first 7 days of PARDS, with the initiation of HFOV occurring on day 2 [1, 3] of PARDS. In our cohort, the median [interquartile range] age was 1.8 [0.5, 6.3] and 2.2 [0.8, 5.3] years for the non-HFOV and HFOV groups, respectively. The HFOV group had the following settings: mean airway pressure 25.0 [20.8, 29.3] cm H_2_O, amplitude 55.0 [46.5, 62.8] and fraction of inspired oxygen 87.9 [71.2, 100] % (Additional file 1: Figure S1). For the non-HFOV group, the breakdown of MV modes was as follows: CMV [165/206 (80.1%)] and airway pressure release ventilation [41/206 (19.9%)]. The settings for those on CMV were peak inspiratory pressure 25.0 [20.0, 28.0] cm H_2_O, end expiratory pressure 7.0 [6.0, 9.0] cm H_2_O, mean airway pressure 14.0 [11.8, 17.2] cm H_2_O, fraction of inspired oxygen 55.0 [40.0, 80.0] % and tidal volume 8.3 [6.6, 10.9] ml/kg. The major causes of PARDS were pneumonia [269/328 (82.0%)] and sepsis [94/328 (28.7%)]. 13/328 (4.0%) patients required ECMO. Compared to the non-HFOV group, the HFOV group had higher OI (18.8 [12.0, 30.2] vs. 7.7 [5.1, 13.1] respectively; *p* < 0.001), increased comorbidities [69/122 (56.6%) vs 93/206 (45.1%); *p* = 0.046] and increased 28-day mortality [38/122 (31.1%) vs 37/206 (18.0%); *p* = 0.007]. From the stratified Cox model, we verified that there was no significant difference in terms of HFOV assignment among the 10 centres. The PS model achieved a fivefold cross validation AUROC of 0.75 for predicting the probability of receiving HFOV. The output from the PS model can be found in the supplementary material (Additional file 1: Table S2).

**Table 1:** Characteristics of patients on high-frequency oscillatory ventilation (HFOV) and non-HFOV before and after genetic matching (GM)

	Original cohort (*n* = 328)			Cohort from Genetic matching (*n* = 236)				
	Non-HFOV (*n* = 206)	HFOV (*n* = 122)	*p* value	SD	Non-HFOV (*n* = 118)	HFOV (*n* = 118)	*p* value	SD
Female gender [*n* (%)]	91 (44.2)	66 (54.1)	0.10	0.20	57 (48.3)	63 (53.4)	0.52	0.10
Age, years (median [IQR])	1.8 [0.5, 6.3]	2.2 [0.8, 5.3)	0.23	0.01	1.9 [0.6, 5.3]	2.3 [0.8, 5.5]	0.25	0.07
PIM 2 (median [IQR])	8.4 [4.1, 16.8]	8.2 [4.7, 27.6]	0.23	0.17	9.4 [5.4, 22.4]	8.0 [4.7, 27.6]	0.76	0.14
PELOD (median [IQR])	7.5 [1.0, 12.0]	10.0 [1.0, 15.8]	0.30	0.14	11.0 [1.0, 13.8]	10.0 [1.2, 16.0]	0.78	0.13
Bacteraemia [*n* (%)]	32 (15.5)	22 (18.0)	0.66	0.07	26 (22)	21 (17.8)	0.51	0.11
MODS [*n* (%)]	82 (39.8)	56 (45.9)	0.33	0.12	59 (50)	54 (45.8)	0.60	0.08
Comorbidity [*n* (%)]	93 (45.1)	69 (56.6)	0.05	0.23	68 (57.6)	66 (55.9)	0.90	0.03
Risk factors for PARDS								
Pneumonia [*n* (%)]	164 (79.6)	105 (86.1)	0.19	0.17	94 (79.7)	102 (86.4)	0.22	0.18
Sepsis [*n* (%)]	61 (29.6)	33 (27.1)	0.71	0.06	43 (36.4)	32 (27.1)	0.16	0.20
Aspiration [*n* (%)]	10 (4.9)	4 (3.3)	0.69	0.08	6 (5.1)	4 (3.4)	0.75	0.08
Transfusion [*n* (%)]	2 (1.0)	3 (2.5)	0.36	0.11	0 (0)	3 (2.5)	0.25	0.23
Trauma [*n* (%)]	4 (1.9)	0 (0)	0.30	0.20	0 (0)	0 (0)	1.00	< 0.01
Drowning [*n* (%)]	9 (4.4)	3 (2.5)	0.55	0.11	2 (1.7)	3 (2.5)	1.00	0.06
Oxygenation index * [*n* (%)]			< 0.001	1.15			0.89	0.10
Mild (4 ≤ OI < 8)	85 (41.3)	10 (8.2)			13 (11.0)	10 (8.5)		
Moderate (8 ≤ OI < 16)	58 (28.2)	35 (28.7)			35 (29.7)	35 (29.7)		
Severe (OI ≥ 16)	39 (18.9)	74 (60.7)			68 (57.6)#	70 (59.3)		

Categorical variables are presented as counts (percentages), continuous variables are presented as median (interquartile range (IQR))

*Taken after 24 h of paediatric acute respiratory distress syndrome diagnosis

#We perform genetic matching with replacement, so some control subject in the non-HFOV may be matched multiple times

*HFOV* high-frequency oscillatory ventilation, *PIM 2* paediatric index of mortality, *PELOD* paediatric logistic organ dysfunction, *MODS* multiorgan dysfunction syndrome, *SD* standardized difference

Using GM, we obtained a balanced cohort with total number of patients *n* = 236 (non-HFOV group *n* = 118 and HFOV group *n* = 118). The cohort was balanced between the non-HFOV and HFOV groups for all covariates in terms of small standardized difference and non-significant *p* values (Table 1). The 28-day mortality for the matched non-HFOV group and HFOV group were 20/118 (16.9%) vs. 38/118 (32.2%); the OR of HFOV was 2.3 (95%CI 1.3–4.4, *p* = 0.01) (Table 2). For secondary outcomes, the VFD was indifferent between the HFOV and non-HFOV groups. The median VFD was 4.0 [0.0, 17.8] days in the non-HFOV group and 4.0 [0.0, 16.0] days in the HFOV group (*p* = 0.29), whereas the IFD was significantly higher in the non-HFOV group. The median IFD was 4.0 [0.0, 15.8] days in the non-HFOV and 0.0 [0.0, 11.0] days in the HFOV group (*p* = 0.03) (Table 2).

**Table 2:** Genetic matching for the primary and secondary outcomes in the non-HFOV and HFOV groups

	Non-HFOV (*n* = 118)	HFOV (*n* = 118)	*p* value	
			McNemar’s test	OR (95%CI)
28-day mortality [*n* (%)]	20 (16.9)	38 (32.2)	0.01	2.3 (1.3, 4.4)
			Kruskal-Wallis test	MD (95% CI)*
VFD (median [IQR])	4.0 [0.0, 17.8]	4.0 [0.0, 16.0]	0.29	−1.3 (−3.4, 0.9)
IFD (median [IQR])	4.0 [0.0, 15.8]	0.0 [0.0, 11.0]	0.03	−2.5 (−4.9, −0.5)

Categorical variables are presented as counts (percentages), continuous variables are presented as median [IQR]

*HFOV* high-frequency oscillatory ventilation, *VFD* 28-day ventilator-free days, *IFD* 28-day intensive care unit-free days, *MD* mean difference, *OR* odds ratio, *95% CI* 95% confidence interval

*VFD and IFD did not follow normal distribution; therefore, we performed non-parametric Kruskal-Wallis test to determine the group differences and calculated the *p* values. Note that we still provide calculations for MD and corresponding 95% CI for VFD and IFD (assuming normal distribution), but the value of MD and 95% CI are only rough references. They should NOT be taken as true estimates and these need to be interpreted with caution

**Fig. 1:**
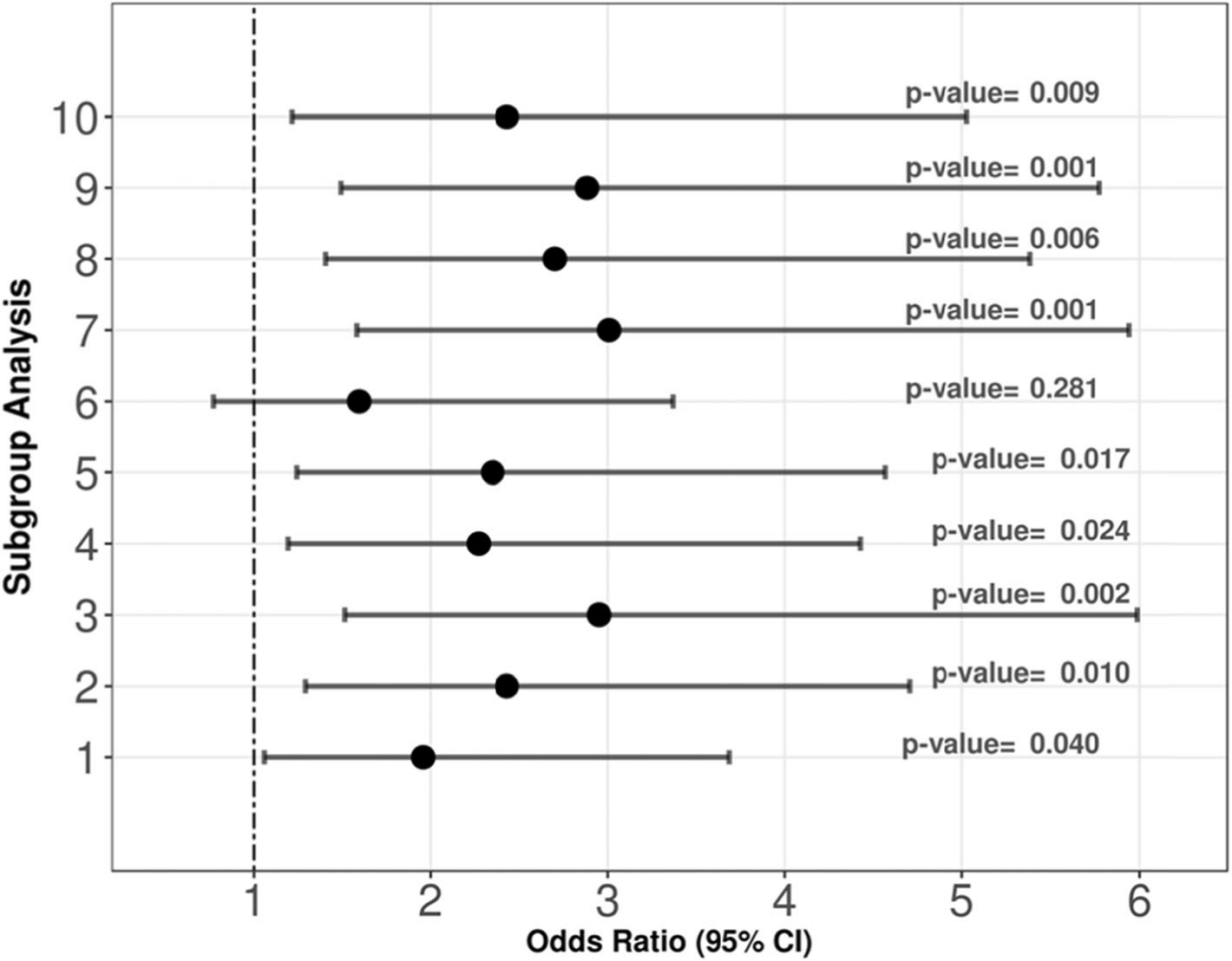
Odds ratio and 95% CI for subgroup analysis. The odds ratio (OR) and 95% CI are represented as black dots and horizontal bars respectively. The subgroup analysis was performed 10 times, while each time exclude one centre from the 10 centres in this study. We observed that the ORs from the 10 experiments were all greater than 1, indicating the 10 centres had consistent harmful outcome of using HFOV in terms of 28-day mortality. The 95% confidence interval of the ORs also support our finding that HFOV was harmful. The *p* values in 9 out of 10 experiments were less than 0.05. By comparing the ORs and 95% CI from the subgroups, we found there was significant association of HFOV treatment with 28-day mortality in PARDS

From the subgroup analysis, GM was robust with different sub-populations as applied in the 10 experiments where the ORs of HFOV towards 28-day mortality were all greater than 1. Concurrently, 9 out of 10 experiments yielded significant *p* values for the ORs (Fig. 1). Further subgroup analysis for age ≥ 1 year vs. age < 1 year, direct vs. indirect PARDS, severe vs. non-severe PARDS and MOD vs. no MOD estimated an OR consistently suggesting a harmful effect for HFOV use with OR > 1 (Additional file 1: Table S3.1 and S3.2). However, the OR showed HFOV was more harmful for certain subgroups (i.e. no MOD), while the effect was less significant for other subgroups (i.e. MOD).

### Sensitivity analysis

The sensitivity analysis performed using three separate statistical approaches: PS matching, IPTW and MSM, showed consistent findings with the primary analysis from the GM approach [28-day mortality OR 1.4 (95% CI 0.6–3.4, *p* = 0.56), 2.1 (95%CI 1.4–3.0; *p* < 0.01) and HR 1.34 (95% CI 0.43–4.14; *p* = 0.61), respectively] (Additional file 1: Table S4, Table S5). The details of the covariate balance and results from PS matching, IPTW and MSM are included in the supplementary material (Additional file 1: Table S6 and SE2-SE4, respectively). Adjustment for time-varying confounding with daily OI during the first week of PARDS (with imputation for missing values) demonstrated consistent direction of effect of the OR in the GM and PSM (Additional file 1: Table S7.1) and the adjusted HR in the MSM (Additional file 1: Table S7.2). Additionally, multivariate logistic regression for 28-day mortality demonstrated a significant harmful effect of using HFOV (Additional file 1: Table S8).

## Discussion

In this study, we evaluated the impact of HFOV use on mortality in children with PARDS by using several different statistical approaches. Data from the original cohort revealed significant differences in baseline OI between the HFOV and non-HFOV groups indicating a tendency to use HFOV in patients with worse oxygenation failure, which was clearly a confounding factor for the estimation of HFOV use on outcomes. By balancing the HFOV and non-HFOV groups with all the confounding factors, all the approaches including GM, PS matching, IPTW and MSM indicated HFOV had potential harmful treatment effect on 28-day mortality, whereas this effect on VFDs and IFDs was less clear.

Our data adds to the limited paediatric data on HFOV use in PARDS. In a retrospective study of 48 children with severe PARDS, when compared to CMV, the use of rescue HFOV was associated with improved gas exchange but not with decreased mortality [18]. The HFOV group had a longer PICU LOS and duration of MV, and vasoactive agent use was more frequent [18]. Another study (*n* = 26) demonstrated increased 30-day survival with the use of early HFOV (within < 24 h) [10/17 (58.8%) vs. 1/9 (12.5%); *p* = 0.01] and suggested that the duration of CMV prior to institution of HFOV influenced HFOV efficacy [23]. Of note, these studies included a limited number of patients and lacked adjustment for relevant covariates (e.g. OI).

The large retrospective study derived from the Virtual PICU System (VPS) database (*n* = 9177) and the post hoc analysis of the Randomized Evaluation of Sedation Titration for Respiratory Failure (RESTORE) study (*n* = 1064), evaluated the use of early (day 1 of intubation) vs. late HFOV using PS matching in children with acute respiratory failure [20, 21]. In comparison to these studies which utilize the PS matching method, our study applies the more robust GM method which achieves covariate balance by direct multivariate matching using an automated search algorithm [29]. Both the VPS and RESTORE re-analysis studies demonstrated increased mortality, duration of MV and PICU stay in the HFOV group. Early use of HFOV compared to late, was also shown to be associated with increased mortality [20]. However, these studies included undifferentiated acute respiratory failure which may consist of patients with less severe hypoxemia compared with PARDS and lack any form of adjustment or matching for granular oxygenation data [55]. It is possible that the results found in these prior studies were due to the inclusion of patients with likely less severe oxygenation deficit who stood to benefit less from HFOV. This postulation is supported by adult data that showed that HFOV was dependent on baseline severity of hypoxemia with harm demonstrated among patients with mild-moderate ARDS, and the possibility of decreased mortality in patients with very severe ARDS [3, 56, 57]. Our subgroup analysis, however, showed consistent harm in the severe group of PARDS, though our analysis is limited by the small number of matched pairs (*n* = 74, Additional file 1: Table S3.1, Table S3.2).

The controversial effects of HFOV on clinical outcomes should also be considered in the context of HFOV-related respiratory and cardiovascular effects. HFOV improves oxygenation by maintaining a higher and more consistent MAP, thereby avoiding conventional swings in airway pressure which increases peak lung stress. The higher airway pressures recruit collapsed regions thereby increasing lung volume and reducing ventilatory strain. Therefore, the main theoretical benefit of HFOV in PARDS is in its ability to prevent volutrauma and atelectrauma which have been shown in clinical trials to worsen outcomes [58, 59]. However, studies using electrical impedance tomography show that some patients recruit unevenly, thereby exposing open regions of lungs to excessively high lung strain [60, 61]. Deleterious hemodynamic effects are also caused by high airway pressures in HFOV and may worsen right ventricular function [62]. Airway pressure-related preload reduction has been demonstrated to occur rapidly after transitioning from CMV to HFOV [63]. These beneficial and harmful effects should be monitored in future trials to better understand the impact of HFOV on clinical outcomes.

This is a relatively large study evaluating HFOV use on mortality in children specifically with PARDS. Advanced statistical methods applying several rigorous matching techniques to assess the stability of results were used to compensate for the lack of randomization and standardized protocol due to the retrospective nature of the study. This study provides a good basis for performing a randomized trial on the effect of HFOV in the setting of PARDS. We estimated the association of HFOV use on mortality using the GM approach and found that HFOV may have a harmful effect. The OSCILLATE trial (*n* = 548) demonstrated a relative risk of death of 1.33 (95% CI 1.09 to 1.64) whereas the OSCAR trial showed no benefit or harm [1.03 (95%CI 0.75 to 1.40)] from the use of HFOV in adults with ARDS. Our study using four statistical approaches revealed a consistent direction of harmful treatment effect on mortality outcome (OR of 1.3–2.3), which indicates significant harm in using HFOV. However, given the limitations of a retrospective study and statistical modelling, one should interpret these results with caution. A conservative conclusion would be that the results of our study suggest caution in the routine use of HFOV the general cohort of children with PARDS.

Other limitations of this study include the use of ventilation data only up to the first 7 days of PARDS diagnosis. Thus, we were only able to adjust for the time-dependent treatment effect and confounding up to the first week in PICU. We also did not include other potentially relevant variables like the PELOD score on the day of switching to HFOV, which may have influenced outcomes. Another limitation was the lack of protocolized MV management in all 10 centres. However, we applied the stratified Cox model to justify that treatment assignments among the 10 centres were indifferent. A randomized trial of HFOV use in PARDS is necessary to address the question of whether the use of HFOV leads to worse clinical outcomes in PARDS and we look forward to the completion of the PROSpect trial (NCT03896763). In addition, studies involving HFOV in PARDS should consider stratification by the severity of illness and include monitoring of hemodynamic and regional lung volumes.

## Conclusion

In PARDS, HFOV use was common, indicating an enduring belief in its advantages despite adult data suggesting harm. With GM and other statistical approaches, we found that HFOV use within the first week of PARDS was also associated with a higher mortality risk. Our study suggests caution but does not reduce equivocality, and a randomized trial is justified to investigate the true effect of HFOV on clinical outcomes in children with PARDS.

## Supplementary information

Additional file 1: Supplementary material. E1. Genetic Matching. E2. Average Propensity Score matching and Daily Propensity Score Matching. E3. Inverse Probability Treatment Weighting. E4. Marginal Structural Model. E4.1. Calculation of Stabilized Weights. E4.2. Calculation of Non-Stabilized Weights. Subgroup Analysis. Analysis adjusting for the time course of PARDS using daily Oxygenation Index. **Table S1.** Characteristics of patients from original cohort and selected cohort **Table S2.** Output from the propensity score model for receiving high frequency oscillatory ventilation **Table S3.1.** Total number of unmatched patients of each subgroup and total matched pairs after genetic matching. **Table S3.2.** Primary and secondary outcomes of each subgroup after genetic matching. **Table S4.** Outcome analysis: HFOV treatment effect with propensity score matching and inverse probability of treatment weighting. **Table S5.** Hazard ratio estimates for HFOV treatment on 28-day mortality from the marginal structural model with stabilized weights. **Table S6.** Characteristics of non-HFOV and HFOV patients before and after adjustment with weights from Inverse Portability Treatment Weighting model and Propensity Score Matching. **Table S7.1.** Primary and secondary outcomes for the non-HFOV and HFOV groups from Genetic Matching and Propensity Score Matching with daily oxygenation index. **Table S7.2.** Hazard ratio for 28-day mortality estimates for HFOV treatment from the Marginal Structural Model with stabilized weights with 24 h OI and daily OI. **Table S8**: Multivariate logistic regression for 28-day mortality. **Figure S1.** Average of daily maximum high frequency oscillatory ventilation settings during the first 7 days of paediatric acute respiratory distress syndrome diagnosis for the original cohort. **Figure S2.** Distribution of (a) log stabilized weights and (b) log non-stabilized weights. **Figure S3.** Validity Check for Marginal Structural Cox model assumption.

## Data Availability

The dataset used and analysed during the current study are available from the corresponding author on reasonable request.

## Abbreviations

ARDS: Acute respiratory distress syndrome
AUROC: Area under the receiver operating characteristic curve
CI: Confidence interval
CMV: Conventional mechanical ventilation
CPH: Cox proportional hazard
GM: Genetic matching
HFOV: High-frequency oscillatory ventilation
HR: Hazard ratio
IFD: Intensive care unit-free days
IPTW: Inverse probability of treatment weighting
MSM: Marginal structural model
MV: Mechanical ventilation
OI: Oxygenation index
OR: Odds ratio
PARDS: Paediatric acute respiratory distress syndrome
PELOD: Paediatric logistic organ dysfunction score
PICU: Paediatric intensive care unit
PIM 2: Paediatric index of mortality 2 score
PS: Propensity score
RCT: Randomized controlled trial
VFD: Ventilator-free days
