# Supplementary material for: The impact of high frequency oscillatory ventilation on mortality in paediatric acute respiratory distress syndrome
Source: Crit Care. 2020 Jan 31;24:31. doi: 10.1186/s13054-020-2741-x (PMC6995130; doi:10.1186/s13054-020-2741-x)
Supplement: Supplementary file 1 — Supplementary material. E1. Genetic Matching. E2. Average Propensity Score matching and Daily Propensity Score Matching. E3. Inverse Probability Treatment Weighting. E4. Marginal Structural Model. E4.1. Calculation of Stabilized Weights. E4.2. Calculation of Non-Stabilized Weights. Subgroup Analysis. Analysis adjusting for the time course of PARDS using daily Oxygenation Index. Table S1. Characteristics of patients from original cohort and selected cohort Table S2. Output from the propensity score model for receiving high frequency oscillatory ventilation Table S3.1. Total number of unmatched patients of each subgroup and total matched pairs after genetic matching. Table S3.2. Primary and secondary outcomes of each subgroup after genetic matching. Table S4. Outcome analysis: HFOV treatment effect with propensity score matching and inverse probability of treatment weighting. Table S5. Hazard ratio estimates for HFOV treatment on 28-day mortality from the marginal structural model with stabilized weights. Table S6. Characteristics of non-HFOV and HFOV patients before and after adjustment with weights from Inverse Portability Treatment Weighting model and Propensity Score Matching. Table S7.1. Primary and secondary outcomes for the non-HFOV and HFOV groups from Genetic Matching and Propensity Score Matching with daily oxygenation index. Table S7.2. Hazard ratio for 28-day mortality estimates for HFOV treatment from the Marginal Structural Model with stabilized weights with 24 h OI and daily OI. Table S8: Multivariate logistic regression for 28-day mortality. Figure S1. Average of daily maximum high frequency oscillatory ventilation settings during the first 7 days of paediatric acute respiratory distress syndrome diagnosis for the original cohort. Figure S2. Distribution of (a) log stabilized weights and (b) log non-stabilized weights. Figure S3. Validity Check for Marginal Structural Cox model assumption. [file 13054_2020_2741_MOESM1_ESM.docx]

**Supplementary Material**

**Contents**

E1: Genetic Matching—page 2

E2: Propensity Score matching and Daily Propensity Score Matching —page 3

E3: Inverse Probability Treatment Weighting —page 4

E4: Marginal Structural Model—page 5

E4.1: Calculation of Stabilized Weights

E4.2: Calculation of Non-Stabilized Weights

Table e1: Characteristics of patients from the original cohort and selected cohort —page 7

Table e2: Output from the propensity score model for receiving high frequency oscillatory ventilation—page 8

Subgroup Analysis —page 9

Table e3.1: Total number of unmatched patients of each subgroup and total matched pairs after genetic matching

Table e3.2: Primary and secondary outcomes of each subgroup after genetic matching

Table e4: Primary and secondary outcomes for HFOV use from Propensity Score Matching and Inverse Probability of Treatment Weighting —page 11

Table e5: Hazard ratio for 28-day mortality estimates for HFOV treatment from the Marginal Structural Model with stabilized weights—page 12

Table e6: Characteristics of non-HFOV and HFOV patients before and after adjustment with weights from Inverse Portability Treatment Weighting model and Propensity Score Matching —page 13

Analysis adjusting for the time course of PARDS using daily Oxygenation Index —page 14

Table e7.1: Primary and secondary outcomes for the non-HFOV and HFOV groups from Genetic Matching and Propensity Score Matching with daily oxygenation index

Table e7.2: Hazard ratio for 28-day mortality estimates for HFOV treatment from the Marginal Structural Model with stabilized weights with 24h OI and daily OI

Table e8: Multivariate logistic regression for 28-day mortality —page 15

Figure e1: Average of daily maximum high frequency oscillatory ventilation settings during the first 7 days of paediatric acute respiratory distress syndrome diagnosis for the original cohort —page 16

Figure e2: Distribution of (a) log stabilized weights and (b) log non-stabilized weights —page 17

Figure e3: Validity Check for Marginal Structural Cox model assumption —page 18

References —page 20

**E1: Genetic Matching**

Genetic matching (GM) automates the process of maximizing balance on observed covariates in the matched sample by using an evolutionary search algorithm to determine the weight each individual covariate is given.[[1](#_ENREF_1)] The key difference between GM and general matching approach, i.e. propensity score matching (PS), is that GM generalize a distance metric and use an iterative search algorithm to maximize covariate balance. Balance is achieved by minimizing the distance metric of the covariates. Mahalanobis distance (MD) is the distance metric between any two samples that can be collapsed into a single scalar.

$MD\left( X_{i}, X_{j} \right)={[\left( X_{i}-X_{j} \right)^{T}S^{-1}\left( X_{i}-X_{j} \right)]}^{1/2}$ (1)

where $S$ is the sample covariance matrix of $X$, and $X^{T}$is the transpose of the matrix X. The PS can be combined with $MD$ by including Pscore a vector in $X$ matrix in (1). GenMatch generalizes the $MD$ by including an additional weight matrix $W$:

$GMD\left( X_{i}, X_{j}, W \right)={[\left( X_{i}-X_{j} \right)^{T}{{(S}^{-\frac{1}{2}})}^{T} W\left( S^{-\frac{1}{2}} \right)\left( X_{i}-X_{j} \right)]}^{1/2}$ (2)

where $W$ is a $k \times k$ positive definite weight matrix with $k$ being the number of covariates, and $S^{-1/2}$ is the Cholesky decomposition of $S$. GM aims to use an iterative search algorithm to minimize the generalized Mahalanobis distance ($GMD$) in (2). By default, $W$ is the diagonal matrix, where each element of the diagonal corresponds to the weight of each covariate. If the weight of the PS is set to zero, then $GMD$ is the same as $MD$. In case of PS misspecification, the search algorithm would gradually reduce the weight of PS to zero. Therefore, GM is robust even if PS is mis-specified.

We only provide a brief idea of conducting the iterative search here. Full details are available in [[2](#_ENREF_2), [3](#_ENREF_3)]. The search algorithm proposes batches of weight, $W$, and moves towards the batch which contain the optimal weights. The optimal weight is determined if the weighted standardized difference is minimized. Each batch is a generation and is used iteratively to produce a subsequent generation with better candidate $W$. The new generation of candidate $W$ evolves towards better $W$ and asymptotically converges to the optimal $W$.

**E2: Propensity Score matching and Daily Propensity Score Matching**

We performed two matching methods with the PS. First, a direct one-to-one matching (calliper 0.01) with *average* PS across the patient’s ICU stay. The average PS was estimated by fitting a logistic regression model for HFOV treatment with all relevant covariates. The output from the PS model is shown in Table e2, where higher the OR (i.e. OR>1) of a covariate indicates the higher probability of a patient to receive the HFOV treatment.

The PS matching approach yielded a balanced cohort of 110 subjects (55 in the HFOV and non-HFOV groups, respectively). After matching, all the covariates are balanced between the two groups in terms of SDs and p-values (Table e5). The 28-day mortality for the matched non-HFOV group and HFOV group were [13/55 (23.6%) vs. 17/55 (30.9%)]. The 28-day mortality OR was 1.4 (95% CI 0.6-3.4, p=0.56). For secondary outcomes, the median VFD was 11.0 [0.0, 21.5] days in the non-HFOV group and 8.0 [0.0, 17.5] days in the HFOV group (p =0.31), while IFD was 3.0 [0.0, 18.0] days in the non-HFOV and 0.0 [0.0, 13.0] days in the HFOV group (p =0.18) (Table e3). Due to insufficient number of matched subjects from PS matching approach, we did not manage to obtain significant result of the treatment effect. However, both primary and secondary outcome from PS matching method showed the direction of the treatment effect were the same as GM.

We then performed an extension to first method by calculating PS for each day of the patient in the ICU, and then perform matching based on PS on a *daily* basis. Daily PS matching is superior than an average PS accounting for the time-varying treatment effect of HFOV. We calculated the stabilized weight (see below) for each patients each day in the ICU. Every patient had changing weights each day across the ICU stay, then the inverse of the stabilized weights were the PS for that patients on each day of his ICU stay. We applied McNemar’s test for primary outcome 28-day mortality. For secondary outcome, we applied non-parametric Kruskal-Wallis test for continuous variable VFD and IFD.

Daily PS matching with calliper 0.01 resulted in total 39 pairs (78 observations). For primary outcome the OR for 28-day mortality was 1.36, 95%CI: (0.44, 4.33) with p-value 0.77. For secondary outcomes, VFD in non-HFOV and HFOV group are (median [IQR]: 15.0 [0.0,22.5] vs. 5.0 [0.0,16.5], p-value 0.01); IFD in non-HFOV and HFOV group are (median [IQR]: 9.0 [0.0,17.5] vs. 0.0 [0.0,11.0], p-value 0.09).

**E3: Inverse Probability Treatment Weighting**

In the Inverse Probability Treatment Weighting (IPTW) model, we first calculated the propensity score (PS), defined as the conditional probability of having the exposure given all covariates. We fitted the logistic regression model to both HFOV and non-HFOV groups to estimate their probability of receiving the HFOV treatment at the time of PARDS diagnosis and marked this probability as PS. The model is shown as following:

$$logit P\left( X=1 | Z \right)=\beta_{0}+\beta_{1}Z_{1}+\beta_{2}Z_{2}+\beta_{3}Z_{3}+\ldots+\beta_{k}Z_{k}$$

In our case, the covariates (Z) included patients’ demographics (age, gender, comorbidities, multiple organ dysfunction), disease severity score (Paediatric Index of mortality 2 [PIM2] score, PELOD), presence of bacteraemia, risk factors for PARDS (e.g., pneumonia, sepsis, aspiration, transfusion, drowning) and oxygenation index (OI) that were taken after 24 hours of PARDS diagnosis [[6-8](#_ENREF_6)]. The inverse of the PS is used to weight the patient cohort for the treatment group and 1/(1-PS) for the control group, thus the term Inverse Probability Treatment Weighting (IPTW). By weighting on the observed data, we were able to get a pseudo-population in which the distributions among the covariates/confounders in the control and treatment groups are the same[[9](#_ENREF_9)]. If the distributions of the covariates are the same in the two groups, then there is no longer association between the covariates and the exposure, making the control and the treatment groups interchangeable [[5](#_ENREF_5)]. Therefore, the crude association between the treatment and the outcome is un-confounded if the PS model is correctly specified and there is no unmeasured confounding.

After the weight adjustment with IPTW, we obtained a weighted cohort with total number of n=688.9 (non-HFOV group n=326.5; HFOV group n=362.4- the numbers of patients were not necessarily integers because weighting can be fractional) (Table e5). The weighted cohort was balanced between the HFOV and non-HFOV groups for all covariates in terms of small standardized difference and non-significant p-values.

Rather than dropping unmatched subjects as PS matching, IPTW makes use of all subjects with weighting. For the 28-day mortality, the OR was 2.1 (95%CI 1.4-3.0; p<0.01). For the secondary outcomes from IPTW, the median VFD was 15.0 [0.0, 23.0] days in the non-HFOV group and 6.6 [0.0, 18.9] days in the HFOV group (p =0.05), while IFD was 10.0 [0.0, 19.0] days in the non-HFOV and 0.0 [0.0, 15.0] days in the HFOV group (p =0.13) (Table e3). IPTW showed the direction of the HFOV treatment effect was consistent with the findings from GM and showed that the use of HFOV was associated with a higher mortality rate and a longer ventilation period compared to patients on conventional ventilations.

**E4: Marginal Structural Model**

Marginal Structural Models (MSM) developed by Robins et al.[[4](#_ENREF_4)] are causal models designed to adjust for time-dependent treatment and confounding in observational studies. In our study, the use of HFOV is a time-dependent exposure, that is one day’s treatment may affect the subsequent days’ treatment. Traditional modelling method will be biased if confounding is not adjusted properly[[5](#_ENREF_5)]. Therefore, we applied MSM in this study and conducted a doubly robust analysis to estimate the causal effect between the time-dependent HFOV treatment and the primary outcome.

This method combines the Cox Proportional Hazards model (CPH model) the IPTW model, thus it is doubly robust. In MSM, we have further considered the time-depended treatment effect of HFOV (during the first 7 days ICU stay) in our IPTW model by modifying the weights to “stabilized” weights [[4](#_ENREF_4), [5](#_ENREF_5), [9](#_ENREF_9)]. Details on the calculation of the stabilized weights are described in the next section.

After calculating the stabilized weights, we constructed the MSM by fitting the CPH model with the “stabilized” weighted cohort to estimate the causal effect of HFOV exposure and our primary outcome. To explain further on the MSM, we borrow the notations from Robin et al[[4](#_ENREF_4)]. We define T to be a patient’s time of death with time measured in days from PICU admission, and we modelled the data till the 28th day in PICU for the primary outcome. Let $A\left( t \right)at t=1$ to be $A_{1}$ , which represents the treatment of HFOV on day 1 in PICU. If the patients receive the HFOV on day 1, then $A_{1}$=1, otherwise 0. And $A_{2}$indicate the exposure to HFOV on day 2 and so on. We have a collection of each patient’s treatment history from day 1 to day 7, (ie. $A_{1}, A_{2},\ldots A_{7}$) and we denote this history as $\bar{A}\left( t \right)=\{A\left( u \right);1\leq u<t\}$. Similarity, we define the measured time-fixed baseline confounding as $L$. In this analysis, we have $L$ as the vector of all the time-fixed covariates as mentioned before (i.e. age, gender, severity score, comorbidity, risk factors, oxygenation index etc.). We denote $\bar{a}\left( t \right)=\{a\left( u \right);1\leq u<t\}$as one possible value of $\bar{A}\left( t \right)$, and let $T_{\overline{a}}$ be the random variable that represent a patient’s time to death if he followed the treatment history of $\bar{a}\left( t \right)$. With that, we propose the time-dependent conditional Cox PH model (a) and marginal structural Cox PH model (b):

$H_{T}(t |\bar{A}\left( t \right), L)=H_{0}\left( t \right) exp( \beta_{1}A\left( t \right)+\beta_{2}L )$ (a)

$H_{T_{\overline{a}}}(t | L)=H_{0}\left( t \right) exp( \beta_{1}^{'}A\left( t \right)+\beta_{2}^{'}L )$ (b)

where in model (a), $H_{T}\left( t | \bar{A}\left( t \right), L \right)$ is the conditional hazard of death at time t given the treatment history $\bar{A}\left( t \right)$ and baseline confounding$L$, and in model (b), $H_{T_{\overline{a}}}(t | L)$ is the hazard of death at time t if all of the subjects (contrary to fact) followed a particular HFOV treatment history of $\bar{a}\left( t \right)$ and with the baseline confounding $L$. We refer to model (b) as MSM because within each level of confounding $L$, it is a causal model for the marginal distribution of the counterfactual variable $T_{\overline{a}}$. Note that the model (b) is different from the model (a) in a way that model (b) is for modelling causal effect for the entire population, while model (a) is conditional on the observed data $\bar{A}\left( t \right)$ and modelling only for the observed associations.

By maximizing the Cox partial likelihood from the conditional CPH model, we can obtain the estimates $\hat{\beta_{1}}, \hat{\beta_{2}}$ of $\beta_{1}$ and $\beta_{2}$ for model (a). However, model (a) is a conditional model and it can only provide these estimates conditional on the observed data, therefore biased to infer the true association of HFOV treatment on mortality for the entire population. According to Robin et al.[[4](#_ENREF_4)], we can eliminate or reduce this bias by modifying model (a) with the contribution of each subject at time t by the stabilized weights. The estimates $\hat{\beta_{1}}, \hat{\beta_{2}}$ from model (a) will converge to $\beta_{1}^{'},\beta_{2}^{'}$ in model (b) with proper adjustment of stabilized weights. In this way, we can (1) estimate the parameters $\hat{\beta_{1}}, \hat{\beta_{2}}$ for the marginal structural Cox model by fitting data to the conditional Cox model with stabilized weights, and (2) we have created a pseudo-population in which we can unbiasedly obtain the crude causal log hazard ratio $\hat{\beta_{1}}$, of HFOV treatment on our primary outcome, and also the hazard ratio $exp(\hat{\beta_{1}})$, its corresponding 95% confidence interval, as well as p-value from the MSM CPH model.

The CPH assumption for the MSM approach was checked using Schoenfeld Residuals Plots (Figure e3). The hazard ratio (HR) for 28-day mortality in the HFOV group for the weighted but unadjusted HR was 1.70 (95% CI 1.06-2.72; p =0.03). Whereas, the weighted and adjusted HR was 1.34 (95% CI 0.43-4.14; p = 0.61). (Table e4). Since MSM was a more complex model and it took into consideration of the time-dependent treatment effect of HFOV with stabilized weights, the model would certainly need more data so that it could have sufficient statistical power to produce significant results. Nevertheless, the estimated HR from MSM showed that the treatment effect was in the same direction as the result from the GM approach.

**E4.1 Calculation of Stabilized Weights**

Same as the notations defined above, we have T to be a patient’s time of death with time measured in days since start of follow up in PICU. Let $A\left( t \right)at t=1$ to be $A_{1}$which represents the treatment of HFOV on day 1 in PICU. and we denote treatment history as $\bar{A}\left( t \right)=\{A\left( u \right);1\leq u<t\}$. Similarity, we define the measured time-fixed baseline confounding as $L$, and let the time-dependent confounding history to be $\bar{C}\left( t \right)=\{C\left( u \right);1\leq u<t\}.$Time fixed confounding does not change over time through the study period, such as gender, age etc. And time-dependent confounding will change over time, such as lab test results etc. We denote $\bar{a}\left( t \right)=\{a\left( u \right);1\leq u<t\}$as one possible value of $\bar{A}\left( t \right)$, and let $T_{\overline{a}}$ be the random variable that represent a patient’s time to death if he followed the treatment history of $\bar{a}\left( t \right)$. Given two models (a) time-dependent conditional CPH model and (b) marginal structural CPH model as described in the last section, we are able to estimate the parameters in model (b) by fitting data to model (a) with stabilized weights.

According to Robins et al[[4](#_ENREF_4)], we can compute the stabilized weights using the following equation for time-dependent treatment and covariates:

$${sw}_{i}\left( t \right) = \prod_{k=1}^{t} \frac{pr\left( A\left( k \right)=a_{i}\left( k \right) \right| \bar{A}\left( k-1 \right)=\bar{a_{i}}\left( k-1 \right), L=l_{i})}{pr\left( A\left( k \right)=a_{i}\left( k \right) \right| \bar{A}\left( k-1 \right)=\bar{a_{i}}\left( k-1 \right), \bar{C}\left( k \right)=\bar{c_{i}}\left( k \right) )}$$

Where t is integer value in days, and we define $\bar{A}(0)$ to be 0. Each factor in the denominator is the conditional probability of a subject receiving treatment at time k given the historical treatment history $\bar{a_{i}}\left( k-1 \right)$ and time-dependent confounding $\bar{c_{i}}\left( k \right)$. Whereas each factor in the numerator is the conditional probability of a subject receiving treatment at time k given the historical treatment history $\bar{a_{i}}\left( k-1 \right)$ and time-fixed confounding $L$, but not further adjusting for the past time-varying confounding history.

**E4.2 Calculation of Non-Stabilized Weights**

“Non-stabilized” weights can be simply calculated by replacing the numerator of the ${sw}_{i}\left( t \right)$ by 1 and include the time-fixed confounding in the denominator. Non-stabilized weights would not affect the consistency of our analysis, but stabilized weights are preferred for their narrower 95% confidence interval. Comparison for stabilized weights and non-stabilized weights can be found in Figure e2.

**Table S1: Characteristics of patients from original cohort and selected cohort**

|  | **Original Cohort n=427** | **Selected Cohort n=328** | **p-value** | | | | **SD** |
| --- | --- | --- | --- | --- | --- | --- | --- |
| Female gender [n (%)] | 207 (48.5) | 157 (47.9) | | | 0.93 | | 0.12 |
| Age, years (median [IQR]) | 1.6 [0.5, 5.5] | 1.9 [0.5, 5.8] | | | 0.35 | | 0.04 |
| PIM 2 (median [IQR]) | 7.0 [3.3, 17.9] | 8.2 [4.3, 19.6] | | | 0.15 | | 0.04 |
| PELOD (median [IQR]) | 10 [1.0, 12.0] | 10 [1.0, 14.2] | | | 0.30 | | 0.07 |
| Bacteraemia [n (%)] | 71 (16.6) | 54 (16.5) | | | 1.00 | | <0.01 |
| MOD [n (%)] | 161 (37.7) | 138 (42.1) | | | 0.25 | | 0.09 |
| Comorbidity [n (%)] | 223 (52.2) | 162 (49.4) | | | 0.48 | | 0.06 |
| Risk factors for PARDS | |  | | |  | |  |
| Pneumonia [n (%)] | 365 (83.4) | 269 (82) | | | 0.69 | | 0.04 |
| Sepsis [n (%)] | 111 (26.0) | 94 (28.7) | | | 0.46 | | 0.06 |
| Aspiration [n (%)] | 23 (5.4) | 14 (4.3) | | | 0.59 | | 0.05 |
| Transfusion [n (%)] | 10 (2.3) | 5 (1.5) | | | 0.59 | | 0.06 |
| Trauma [n (%)] | 4 (0.9) | 4 (1.2) | | | 0.99 | | 0.03 |
| Drowning [n (%)] | 14 (3.3) | 12 (3.7) | | | 0.93 | | 0.02 |
| Oxygenation index * | |  | | 1.00 | | | <0.01 |
| Mild (4≤OI<8) [n (%)] | 95 (29.0) | 95 (29) | |  | |  | |
| Moderate(8≤OI<16) [n (%)] | 93 (28.4) | 93 (28.4) | |  | |  | |
| Severe (OI≥16) [n (%)] | 113 (34.5) | 113 (34.5) | |  | |  | |

Categorical variables are presented as counts (percentages), continuous variables are presented as median (interquartile range (IQR))

*Taken after 24hours of paediatric acute respiratory distress syndrome diagnosis

PIM 2: Paediatric Index of Mortality; PELOD: Paediatric Logistic Organ Dysfunction;

MOD: Multiorgan Disfunction; SD: Standardized Difference.

**Table S2: Output from the propensity score model for receiving high frequency oscillatory ventilation**

|  | **Odds Ratio** | **95% Confidence interval** | | **p-value** |
| --- | --- | --- | --- | --- |
| Female gender | 1.48 | 0.85 | 2.58 | 0.17 |
| Age, years | 0.99 | 0.93 | 1.05 | 0.80 |
| PIM 2 | 1.01 | 0.99 | 1.02 | 0.53 |
| PELOD | 1.03 | 0.99 | 1.06 | 0.12 |
| Bacteraemia | 1.54 | 0.69 | 3.41 | 0.29 |
| Multiorgan dysfunction | 0.82 | 0.45 | 1.51 | 0.53 |
| Comorbidities | 1.10 | 0.62 | 1.94 | 0.75 |
| Risk factor for PARDS |  |  |  |  |
| Pneumonia | 1.66 | 0.69 | 3.99 | 0.25 |
| Sepsis | 0.48 | 0.24 | 0.99 | 0.05 |
| Aspiration | 0.94 | 0.24 | 3.66 | 0.93 |
| Transfusion | 9.93 | 1.07 | 92.05 | 0.04 |
| Drowning | 0.87 | 0.13 | 5.66 | 0.88 |
| Oxygenation index* |  |  |  |  |
| Moderate (8≤OI<16) | 6.06 | 2.75 | 14.44 | <0.001 |
| Severe (OI≥16) | 21.02 | 9.55 | 50.84 | <0.001 |

*Taken after 24hours of PARDS diagnosis; reference group is mild PARDS

PARDS: Paediatric acute respiratory distress syndrome

PELOD: Paediatric Logistic Organ Dysfunction score

PIM 2: Paediatric Index of Mortality 2 score

**Subgroup analysis**

**Table S3.1: Total number of patients of each subgroup (Pre-matching) and total matched pairs after genetic matching**

| **Subgroup** | | **Pre-matching** | | | **Matched pairs (n)** | |
| --- | --- | --- | --- | --- | --- | --- |
|  |  | **Non-HFOV**  **n=206** | | **HFOV**  **n=122** |  |  |
|  | Age≥1 year | | 119 (57.8) | 86 (70.5) | 84 |  |
|  | Age<1 year | | 87 (42.2) | 36 (29.5) | 28 |  |
|  | Direct PARDS | | 177 (85.9) | 108 (88.5) | 101 |  |
|  | Indirect PARDS | | 29 (14.1) | 14 (11.5) | 11 |  |
|  | Severe PARDS | | 39 (18.9) | 74 (60.7) | 74 |  |
|  | Non-severe PARDS | | 167 (81.1) | 48 (39.3) | 48 |  |
|  | MOD | | 82 (39.8) | 56 (45.9) | 54 |  |
|  | No-MOD | | 124 (60.2) | 66 (54.1) | 61 |  |

Categorical variables are presented as counts (percentages)

Severe PARDS: 24h oxygenation index >=16

HFOV: High frequency oscillatory ventilation; PARDS: pediatric acute respiratory distress syndrome

MOD: Multi-organ dysfunction

**Table S3.2: Primary and secondary outcomes of each subgroup after genetic matching**

| **Outcome** | | **Subgroup** | | | | **Non-HFOV** | | **HFOV** | | | **p-value** | | | | | |  | |
| --- | --- | --- | --- | --- | --- | --- | --- | --- | --- | --- | --- | --- | --- | --- | --- | --- | --- | --- |
|  |  | | | |  | | |  | **McNemar's Test** | | | | | | | | | **OR (95%CI)** |
| 28-day mortality [n (%)] | | |  | |  | | |  | | |  | |  | | | | | |
|  | Age>=1 | | | | 14 (16.7) | | | 25 (29.8) | | | 0.06 | | | | | 2.1 (1.0,4.5) | | |
|  | Age<1 | | | | 4 (14.3) | | | 10 (37.5) | | | 0.07 | | | | | 3.2 (0.9, 13.8) | | |
|  | Direct PARDS | | | | 22 (21.8) | | | 35 (34.7) | | | 0.05 | | | | | 1.9 (1.0, 3.6) | | |
|  | Indirect PARDS | | | | 1 (9.1) | | | 2 (18.2) | | | 1.00 | | | | | 2.0 (0.1, 71.7) | | |
|  | Severe PARDS | | | | 14 (18.9) | | | 20 (27.0) | | | 0.29 | | | | | 1.6 (0.7, 3.5) | | |
|  | Non-severe PARDS | | | | 8 (16.7) | | | 18 (37.5) | | | 0.06 | | | | | 2.9 (1.1, 8.1) | | |
|  | MOD | | | | 15 (27.8) | | | 19 (35.2) | | | 0.56 | | | | | 1.4 (0.6, 3.2) | | |
|  | No MOD | | | | 4 (6.6) | | | 17 (27.9) | | <0.01 | | | | | | 5.3 (1.8, 20.0) | | |
|  |  | | | |  | | |  | **Kruskal-Wallis Test** | | | | | | | | **MD (95% CI) *** | |
| VFD (median [IQR]) | |  | | |  | | |  |  | | | | | | | |  | |
|  | Age≥1 | | | | 7.0 [0.0, 15.0] | | | 5.0 [0.0, 16.0] | | | 0.80 | | | | -0.3 (-2.7, 2.2) | | | |
|  | Age<1 | | | | 15.0 [0.0, 20.0] | | | 0.0 [0.0, 16.0] | | | 0.04 | | | | -5.6 (-9.5, -1.8) | | | |
|  | Direct PARDS | | | | 10.0 [0.0, 19.0] | | | 4.0 [0.0, 16.0] | | | 0.12 | | | | -1.9 (-4.2, 0.4) | | | |
|  | Indirect PARDS | | | | 0.0 [0.0, 14.0] | | | 0.0 [0.0, 18.5] | | | 0.91 | | | | 0.8 (-8.6, 10.2) | | | |
|  | Severe PARDS | | | | 7.0 [0.0, 13.0] | | | 0.0 [0.0, 13.8] | | | 0.07 | | | | -0.6 (-3.1, 1.9) | | | |
|  | Non-severe PARDS | | | | 18.0 [1.8, 21.0] | | | 6.5 [0.0, 16.2] | | | | <0.01 | | | -4.9 (-8.1, -1.6) | | | |
|  | MOD | | | | 0.0 [0.0, 4.5] | | | 0.0 [0.0, 11.8] | | | | 0.45 | | | 1.3 (-1.8, 4.4) | | | |
|  | No MOD | | | 15.0 [10.0, 20.0] | | | 11.0 [0.0, 18.0] | | | | | 0.01 | | | -4.1 (-6.9, -1.2) | | | |
| IFD (median [IQR]) | |  | | |  | | |  | | | |  | | |  | | | |
|  | Age≥1 | | | | 4.0 [0.0, 12.5] | | | 0.0 [0.0, 11.0] | | | 0.30 | | -1.0 (-3.4, 1.3) | | | | | |
|  | Age<1 | | | | 14.5 [0.0, 20.2] | | | 0.0 [0.0, 15.0] | | | 0.02 | | -5.7 (-9.1, -2.2) | | | | | |
|  | Direct PARDS | | | | 4.0 [0.0, 17.0] | | | 0.0 [0.0, 11.0] | | | 0.06 | | -2.5 (-4.6, -0.3) | | | | | |
|  | Indirect PARDS | | | | 0.0 [0.0, 10.5] | | | 0.0 [0.0, 11.0] | | | 0.63 | | -1.1 (-8.5, 6.3) | | | | | |
|  | Severe PARDS | | | | 2.0 [0.0, 8.5] | | | 0.0 [0.0, 10.5] | | | 0.04 | | -0.3 (-2.5, 1.9) | | | | | |
|  | Non-severe PARDS | | | | 15.5 [0.0, 18.5] | | | 0.0 [0.0, 14.2] | | | <0.01 | | | -5.4 (-8.4, -2.5) | | | | |
|  | MOD | | | | 0.0 [0.0, 0.8] | | | 0.0 [0.0, 4.2] | | | 0.85 | | -0.1 (-2.8, 2.5) | | | | | |
|  | No MOD | | | | 13.0 [4.0, 19.0] | | | 4.0 [0.0, 15.0] | | | <0.01 | | | -4.2 (-7.0, -1.5) | | | | |

Categorical variables are presented as counts (percentages), continuous variables are presented as median [IQR]

HFOV – High frequency oscillatory ventilation; VFD:28-day ventilator-free days;

IFD: 28-day intensive care unit-free days; MD: Mean difference;

OR: Odds Ratio; 95% CI: 95 percent confidence interval.

PARDS: pediatric acute respiratory distress syndrome MOD: Multi-organ dysfunction

*VFD and IFD did not follow normal distribution, therefore we performed non-parametric Kruskal-Wallis test to determine the group differences and calculated the p-values. Note that we still provide calculations for MD and corresponding 95% CI for VFD and IFD (assuming normal distribution), but the value of MD and 95% CI are only for rough references. They should NOT be taken as true estimates and these need to be interpreted with caution.

**Table S4: Primary and secondary outcomes for HFOV use from Propensity Score Matching and Inverse Probability of Treatment Weighting**

| **Outcome** | **Method** | **Non-HFOV** | **HFOV** | | **p-value** | |  | |
| --- | --- | --- | --- | --- | --- | --- | --- | --- |
|  |  |  |  | **McNemar's Test** | | | | **OR (95%CI)** |
| 28-day mortality [n (%)] |  |  |  | |  |  | | |
|  | PS | 13 (23.6) | 17 (30.9) | | 0.56 | 1.4 (0.6, 3.4) | | |
|  | IPTW | 62 (19.0) | 121 (33.3) | | <0.01 | 2.1 (1.4, 3.0) | | |
|  |  |  |  | **Kruskal-Wallis Test** | | | **MD (95% CI) *** | |
| VFD (median [IQR]) |  |  |  | |  | |  | |
|  | PS | 11.0 [0.0, 21.5] | 8.0 [0.0, 17.5] | | 0.31 | -0.9 (-5.5, 2.2) | | |
|  | IPTW | 15.0 [0.0, 23.0] | 6.6 [0.0, 18.9] | | 0.05 | -3.3 (-7.2, 0.6) | | |
| IFD (median [IQR]) |  |  |  | |  |  | | |
|  | PS | 3.0 [0.0, 18.0] | 0.0 [0.0, 13.0] | | 0.18 | -1.7 (-5.9, 0.5) | | |
|  | IPTW | 10.0 [0.0, 19.0] | 0.0 [0.0, 15.0] | | 0.13 | -3.2 (-7.3, 0.9) | | |

Categorical variables are presented as counts (percentages), continuous variables are presented as median [IQR]

HFOV: High frequency oscillatory ventilation; VFD:28-day ventilator-free days;

IFD: 28-day intensive care unit-free days; PS: Propensity score matching;

IPTW: inverse probability of treatment weighting MD: Mean difference;

OR: odds ratio; CI: confidence interval.

*VFD and IFD did not follow normal distribution, therefore we performed non-parametric Kruskal-Wallis test to determine the group differences and calculated the p-values. Note that we still provide calculations for MD and corresponding 95% CI for VFD and IFD (assuming normal distribution), but the value of MD and 95% CI are only for rough references. They should NOT be taken as true estimates and these need to be interpreted with caution.

**Table S5: Hazard ratio for 28-day mortality estimates for HFOV treatment from the Marginal Structural Model with stabilized weights**

|  | **Hazard Ratio** | | | **95% Confidence Interval** | **p-value** |
| --- | --- | --- | --- | --- | --- |
| Stabilized weights | |  |  | |  |
| un-adjusted | | 1.70 | (1.06, 2.72) | | 0.03 |
| adjusted | | 1.34 | (0.43, 4.14) | | 0.61 |

Stabilized weights: Stabilized weights are calculated to incorporate time-dependent treatment effect. The detailed explanations and calculation for stabilized weights are covered in supplementary material E4.1

un-adjusted: The MSM only include HFOV as the variable in the Cox Proportional Hazard model and does not include other confounding factors as covariates.

adjusted: The MSM include both HFOV and all other confounding factors as covariates.

**Table S6: Characteristics of non-HFOV and HFOV patients before and after adjustment with weights using the Inverse Portability Treatment Weighting model and Propensity Score Matching**

| **Patient Characteristics** | | **Original Cohort (n=328)** | | | | | | | | | | | | **Weighted Cohort with IPTW**  **(n=688.9)** | | | | | | | | | | | | | **Cohort after PS matching (n=110)** | | | | | | | | | | | | | |  |
| --- | --- | --- | --- | --- | --- | --- | --- | --- | --- | --- | --- | --- | --- | --- | --- | --- | --- | --- | --- | --- | --- | --- | --- | --- | --- | --- | --- | --- | --- | --- | --- | --- | --- | --- | --- | --- | --- | --- | --- | --- | --- |
|  | **Non-HFOV n=206** | | | | **HFOV n=122** | **p-value** | | | | **SD** | | | | | **Non-HFOV**  **n=326.5** | | **HFOV**  **n=362.4** | | **p-value** | | | | **SD** | | | **Non-HFOV n=55** | | **HFOV n=55** | | | **p-value** | | | | | | **SD** | | | |  |
| Female gender [n (%)] | | | 91 (44.2) | | 66 (54.1) | | | 0.10 | | | 0.20 | | | | 159.5 (48.8) | | 139.4 (38.5) | | 0.22 | | | | 0.21 | | | 31 (56.4) | | | 27 (49.1) | | | 0.57 | | | | | | | 0.15 | |  |
| Age (median [IQR]) | | | 1.8 [0.5, 6.3] | | 2.2 [0.8, 5.3) | | | 0.23 | | | 0.01 | | | | 1.8 [0.5, 5.8] | | 1.8 [0.3, 5.9] | | 0.91 | | | | 0.11 | | | 1.3 [0.5, 7.0] | | | 2.1 [0.7, 4.6] | | | 0.63 | | | | | | | 0.16 | |  |
| PIM 2 (median [IQR]) | | | 8.4 [4.1, 16.8] | | 8.2 [4.7, 27.6] | | | 0.23 | | | 0.17 | | | | 9.2 [4.1, 18.5] | | 6.8 [2.7, 26.6] | | 0.70 | | | | 0.01 | | | 9.2 [3.7, 16.8] | | | 9.0 [5.8, 28.1] | | | | | 0.27 | | | | | 0.14 | |  |
| PELOD (median [IQR]) | | | 7.5 [1.0, 12.0] | | 10.0 [1.0, 15.8] | | | 0.30 | | | 0.14 | | | | 10.0 [1.0, 13.0] | | 10.0 [2.0, 15.1] | | 0.69 | | | | 0.02 | | | 10.0 [1.0, 20.0] | | | 10.0 [1.0, 15.5] | | | | | 0.84 | | | | | 0.06 | |  |
| Bacteraemia [n (%)] | | | 32 (15.5) | | 22 (18.0) | | | 0.66 | | | 0.07 | | | | 51.3 (15.7) | | 46.1 (12.7) | | 0.52 | | | | 0.09 | | | 11 (20.0) | | | 10 (18.2) | | | | 1.00 | | | | | | | 0.05 | |
| MOD [n (%)] | | | 82 (39.8) | | 56 (45.9) | | | 0.33 | | | 0.12 | | | | 137.5 (42.1) | | 151.2 (41.7) | | 0.97 | | | | 0.01 | | | 32 (58.2) | | | 24 (43.6) | | | | 0.18 | | | | | | | 0.29 | |
| Comorbidity [n (%)] | | | 93 (45.1) | | 69 (56.6) | | | 0.05 | | | 0.23 | | | | 163.8 (50.2) | | 162.7 (44.9) | | 0.56 | | | | 0.11 | | | 27 (49.1) | | | 31 (56.4) | | | | 0.57 | | | | | | | 0.15 | |
| Risk factors for PARDS | | | | |  | | |  | | | | |  | |  | | |  |  | | | | |  | |  | | | |  | |  | | |  | | | | | |  |
| Pneumonia [n (%)] | | | | | 164 (79.6) | 105 (86.1) | | | 0.19 | | | | 0.17 | | | 268.5 (82.2) | 255.5 (70.5) | | | 0.23 | | | 0.28 | | | | 41 (74.5) | | | | 44 (80.0) | | 0.65 | | | | | | | 0.13 | |
| Sepsis [n (%)] | | | | | 61 (29.6) | 33 (27.1) | | | 0.71 | | | | 0.06 | | | 93.1 (28.5) | 139.8 (38.6) | | | 0.31 | | | 0.21 | | | | 17 (30.9) | | | | 17 (30.9) | | 1.00 | | | | | <0.001 | | | |
| Aspiration [n (%)] | | | | | 10 (4.9) | 4 (3.3) | | | 0.69 | | | | 0.08 | | | 13.8 (4.2) | 9.1 (2.5) | | | 0.39 | | | 0.10 | | | | 1 (1.8) | | | | 3 (5.5) | | 0.61 | | | | | | | 0.20 | |
| Transfusion [n (%)] | | | | | 2 (1.0) | 3 (2.5) | | | 0.36 | | | | 0.11 | | | 3.1 (1.0) | 4.5 (1.2) | | | 0.79 | | | 0.03 | | | | 0 (0) | | | | 1 (1.8) | | 1.00 | | | | | | | 0.19 | |
| Trauma [n (%)] | | | | | 4 (1.9) | 0 (0) | | | 0.30 | | | | 0.20 | | | 0 (0) | 0 (0) | | | 1.00 | | <0.001 | | | | | 0 (0) | | | | 0 (0) | | 1.00 | | | | | <0.001 | | | |
| Drowning [n (%)] | | | | | 9 (4.4) | 3 (2.5) | | | 0.55 | | | | 0.11 | | | 11.1 (3.4) | 7.5 (2.1) | | | 0.49 | | | 0.08 | | | | 2 (3.6) | | | | 3 (5.5) | | 1.00 | | | | | | | 0.09 | |
| Oxygenation index * [n (%)] | | | | |  | | <0.001 | | | | | | 1.15 | |  | | |  | 0.67 | | | | 0.20 | | |  | |  | | | | 0.11 | | | | | | | 0.48 | |  |
| Mild (4≤OI<8) | | | | | 85 (41.3) | 10 (8.2) | |  | |  | | | | | | 94.8 (29.0) | 138.2 (38.1) | | | |  | |  | | | | 9 (16.4) | | 7 (12.7) | | | |  | | | |  | | | | |
| Moderate(8≤OI<16) | | | | | 58 (28.2) | 35 (28.7) | |  | |  | | | | | | 91.8 (28.1) | 87.5 (24.2) | | | |  | |  | | | | 16 (29.1) | | 23 (41.8) | | | |  | | | |  | | | | |
| Severe (OI≥16) | | | | | 39 (18.9) | 74 (60.7) | |  | |  | | | | | | 112.8 (34.6) | 113.9 (31.4) | | | |  | | | | |  | 23 (41.8) | | 24 (43.6) | | | |  | | | | | |  | | |

Categorical variables are presented as counts (percentages), continuous variables are presented as median (interquartile range (IQR))

*Taken after 24hours of paediatric acute respiratory distress syndrome diagnosis

IPTW: Inverse Portability Treatment Weighting; HFOV: High frequency oscillatory ventilation;

PIM 2: Paediatric Index of Mortality 2 score; PELOD: Paediatric Logistic Organ Dysfunction score;

MOD: Multiorgan dysfunction; OI: oxygenation index; SD: Standardized Difference

**Analysis adjusting for the time course of PARDS using daily Oxygenation Index**

**Table S7.1: Primary and secondary outcomes for the non-HFOV and HFOV groups from Genetic Matching and Propensity Score Matching with daily oxygenation index**

| **Outcome** | **Method** | **Non-HFOV** | **HFOV** | | **p-value** | |  | |
| --- | --- | --- | --- | --- | --- | --- | --- | --- |
|  |  |  |  | **McNemar's Test** | | | | **OR (95%CI)** |
| 28-day mortality [n (%)] |  |  |  | |  |  | | |
|  | GM | 16 (14.1) | 30 (26.5) | | 0.03 | 2.16 (1.11, 4.37) | | |
|  | PSM | 19 (20.2) | 25 (26.6) | | 0.32 | 1.43 (0.72, 2.85) | | |
|  |  |  |  | **Kruskal-Wallis Test** | | | **MD (95% CI) *** | |
| VFD (median [IQR]) |  |  |  | |  | |  | |
|  | GM | 10.0 [0.0, 20.0] | 4.0 [0.0, 16.0] | | 0.04 | -2.2 (-4.7, 0.2) | | |
|  | PSM | 13.0 [0.0, 19.0] | 5.0 [0.0, 16.0] | | 0.07 | -1.7 (-4.7, -0.2) | | |
| IFD (median [IQR]) |  |  |  | |  |  | | |
|  | GM | 3.0 [0.0, 17.0] | 0.0 [0.0, 11.0] | | 0.06 | -2.4 (-4.4, -0.4) | | |
|  | PSM | 5.0 [0.0, 15.0] | 0.0 [0.0, 11.0] | | 0.07 | -2.2 (-4.6, -0.2) | | |

Categorical variables are presented as counts (percentages), continuous variables are presented as median [IQR]

GM: genetic matching PSM: propensity score matching

HFOV: High frequency oscillatory ventilation; VFD:28-day ventilator-free days;

IFD: 28-day intensive care unit-free days; MD: Mean difference;

OR: Odds Ratio; 95% CI: 95 percent confidence interval.

*VFD and IFD did not follow normal distribution, therefore we performed non-parametric Kruskal-Wallis test to determine the group differences and calculated the p-values. Note that we still provide calculations for MD and corresponding 95% CI for VFD and IFD (assuming normal distribution), but the value of MD and 95% CI are only for rough references. They should NOT be taken as true estimates and these need to be interpreted with caution.

**Table S7.2: Hazard ratio for 28-day mortality estimates for HFOV treatment from the Marginal Structural Model with stabilized weights with 24h OI and daily OI**

|  | **Hazard Ratio** | | | **95% Confidence Interval** | **p-value** |
| --- | --- | --- | --- | --- | --- |
| Stabilized weight with adjustment | |  |  | |  |
| 24h OI | | 1.34 | (0.43, 4.14) | | 0.61 |
| Daily OI | | 1.25 | (0.57, 2.74) | | 0.57 |

Stabilized weights with adjustment: Stabilized weights are calculated to incorporate time-dependent treatment effect together with the confounding factors as covariates in the MSM.

24h OI: static oxygenation index measured at the 24h of PARDS

Daily OI: time-varying oxygenation index measured daily during the PARDS course, missing values were imputed with each patient’s last known OI value.

**With daily OI (missing with imputation) in the MSM, the 95% confidence interval is narrower compared with the one using the 24h OI.**

**Table S8: Multivariate logistic regression for 28-day mortality**

|  | $\boldsymbol{\beta}$ | | **Odds Ratio** | **95% CI** | | **p-value** |
| --- | --- | --- | --- | --- | --- | --- |
| **HFOV** | **0.79** | | **2.20** | **1.14** | **4.33** | **0.02** |
| Female | 0.39 | | 1.48 | 0.83 | 2.68 | 0.19 |
| Age (year) | -0.01 | | 0.99 | 0.93 | 1.06 | 0.82 |
| PIM 2 | 0.01 | | 1.00 | 0.98 | 1.01 | 0.55 |
| PELOD | 0.04 | | 1.04 | 1.00 | 1.07 | 0.04 |
| Bacteraemia | 0.92 | | 2.52 | 1.17 | 5.38 | 0.02 |
| MOD | 1.30 | | 3.66 | 1.90 | 7.23 | 0.00 |
| Comorbidity | -0.14 | | 0.87 | 0.47 | 1.57 | 0.64 |
| Risk factor for PARDS: |  | |  |  |  |  |
| Pneumonia | 0.19 | | 1.20 | 0.50 | 3.02 | 0.68 |
| Sepsis | -0.43 | | 0.65 | 0.30 | 1.37 | 0.27 |
| Aspiration | -0.53 | | 0.59 | 0.08 | 2.61 | 0.53 |
| Transfusion | 1.19 | | 3.27 | 0.35 | 26.16 | 0.26 |
| Drowning | 0.02 | | 1.02 | 0.17 | 5.48 | 0.99 |
| Oxygenation index (OI)*: | |  |  |  |  |  |
| Mild (4≤OI<8) | 0.48 | | 1.61 | 0.49 | 6.39 | 0.46 |
| Moderate (8≤OI<16) | 0.02 | | 1.02 | 0.30 | 4.07 | 0.98 |
| Severe (OI≥16) | 0.003 | | 1.00 | 0.29 | 4.08 | 1.00 |

*Taken after 24hours of pediatric acute respiratory distress syndrome diagnosis;

HFOV: high frequency oscillatory ventilation; PIM 2: Pediatric Index of Mortality 2 score;

PELOD: Pediatric Logistic Organ Dysfunction; MOD: multiorgan dysfunction;

PARDS: pediatric acute respiratory distress syndrome.

**Figure S1: Average of daily maximum high frequency oscillatory ventilation settings during the first 7 days of paediatric acute respiratory distress syndrome diagnosis for the original cohort**

MAP: mean airway pressure

FiO2: fraction of inspired oxygen

The HFOV group had the following settings (median [IQR]): mean airway pressure 25.0 [20.8, 29.3] cm H_2_O, amplitude 55.0 [46.5, 62.8] and fraction of inspired oxygen 87.9 [71.2, 100] %.


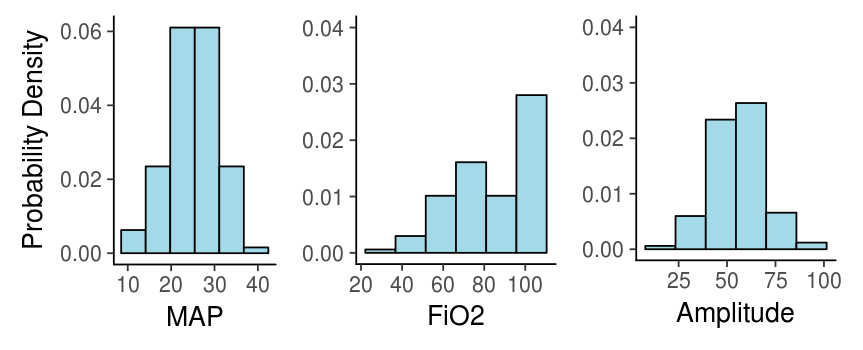


**Figure S2.** **Distribution of (a) log stabilized weights and (b) log non-stabilized weights**

**
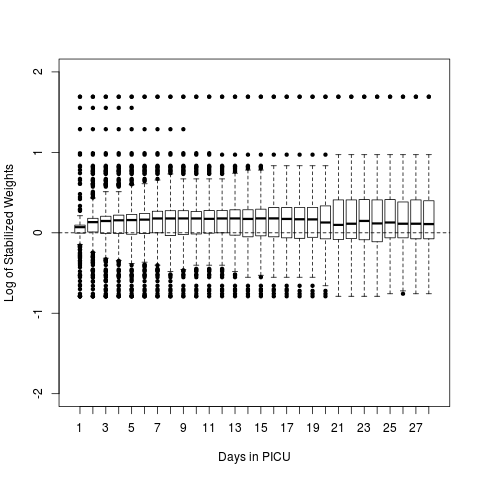

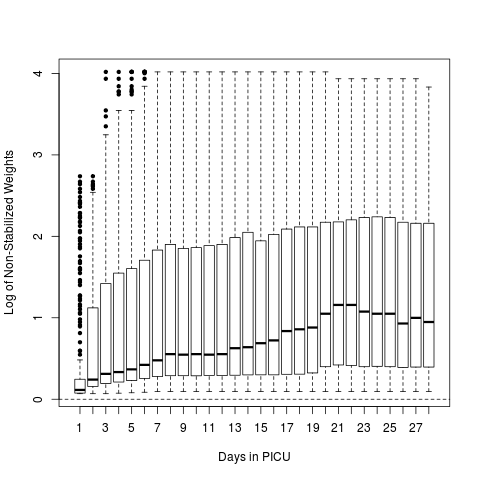
**

**(a) log stabilized (b) log non-stabilized weights**

Boxplots representing the mean (bold horizontal bar) and quantiles (rectangular boxes) of the weights from MSM model for the corresponding day in the paediatric intensive care unit. The observations beyond the 1.5 interquartile range (vertical dashed lines) are plotted individually as dots.

**Comparison of stabilized weights and Non-stabilized weights**

The distribution of log “stabilized” weights was symmetric and centred around 0, while the interquartile range (IQR) broaden over time, whereas the distribution of the “non-stabilized” weights was skewed and the IQR was much wider (Figure e2). This indicates that results using “stabilized” weights are robust even with strong confounding.

From the two plots, we can see the distribution of log stabilized weights (Figure e2a) was symmetric and cantered around 0, while the interquartile range (IQR) broaden over time. Whereas the distribution of the non-stabilized weights (Figure e2b) was skewed and the range of IQR was much wider than the IQR of stabilized weight. Non-stabilized weights would not affect the consistency of our analysis, but stabilized weights are preferred for their narrower 95% confidence interval. We applied stabilized weights for each patient-day in the MSM Cox PH model, and this is to ensure the robustness of our finding for the causal relation between HFOV use and 28-day mortality.

**Figure S3: Validity Check for Marginal Structural Cox model assumption**

**Schoenfeld Residuals Plots**

**
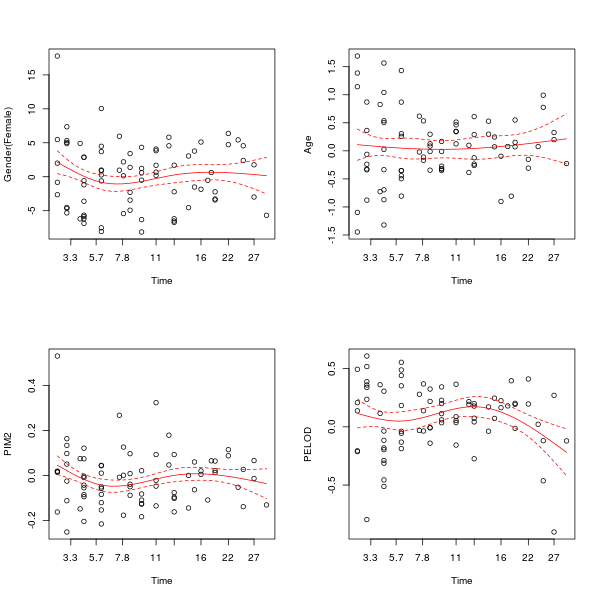

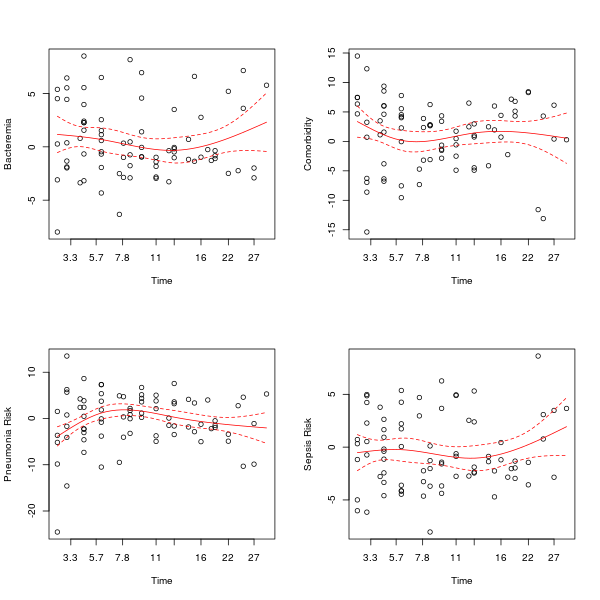
**

**
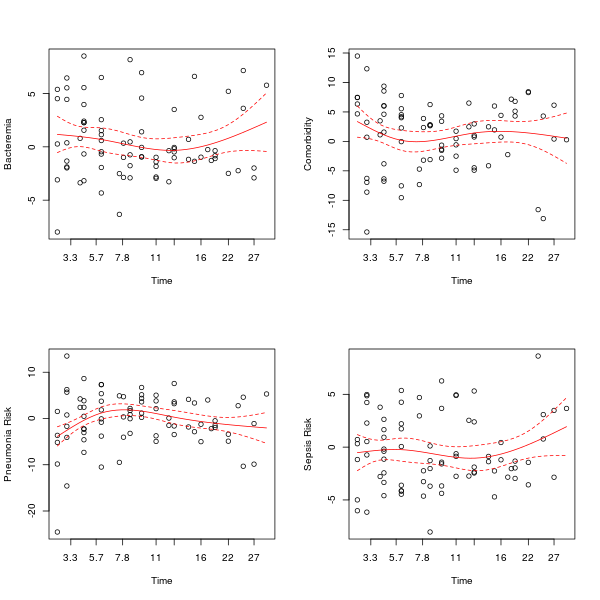

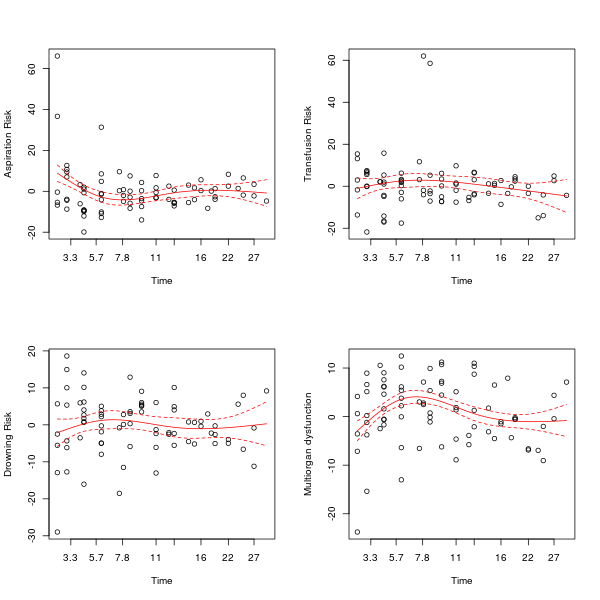
**

**
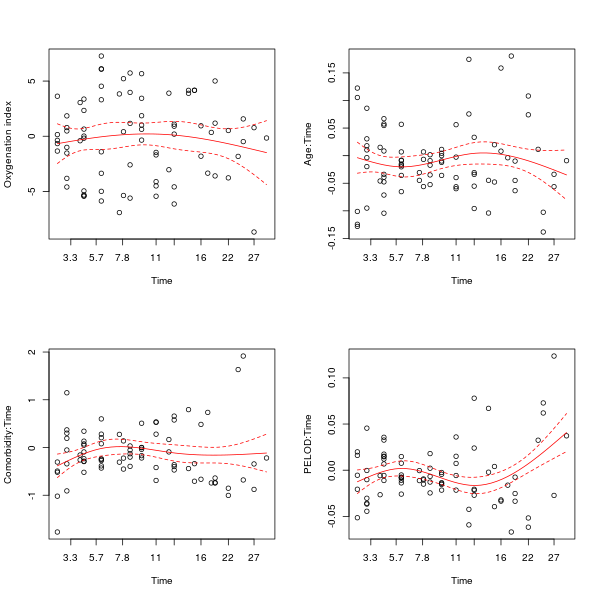
**

**
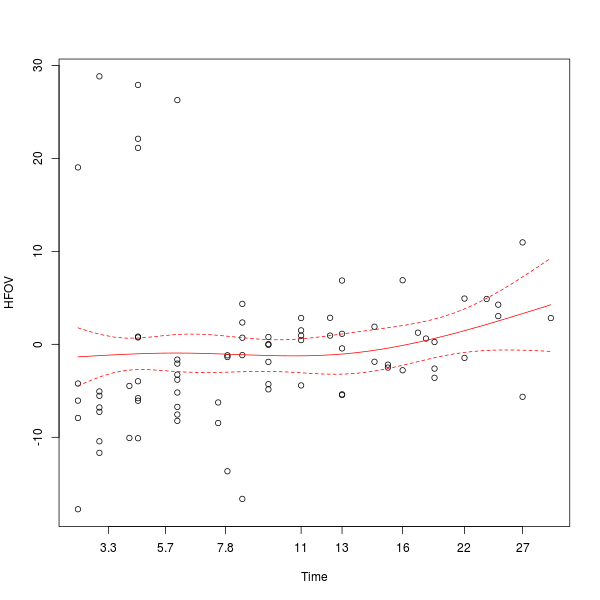
**

**Schoenfeld Residuals (SR) plots of exposure variable HFOV, confounding variables and interaction terms for the MSM. The plots show SR of all the variables are flat over time and this indicates a proportional hazard for the MSM CPH Model.**

**References**

1. Radice, R., et al., *Evaluating treatment effectiveness in patient subgroups: a comparison of propensity score methods with an automated matching approach.* Int J Biostat, 2012. **8**(1): p. 25.

2. Sekhon, J.S., *Multivariate and propensity score matching software with automated balance optimization: the matching package for R.* Journal of Statistical Software, Forthcoming, 2008.

3. Mebane Jr, W.R. and J.S. Sekhon, *Genetic optimization using derivatives: the rgenoud package for R.* Journal of Statistical Software, 2011. **42**(11): p. 1-26.

4. Robins, J.M., M.A. Hernan, and B. Brumback, *Marginal structural models and causal inference in epidemiology.* Epidemiology, 2000. **11**(5): p. 550-60.

5. Funk, M.J., et al., *Doubly robust estimation of causal effects.* Am J Epidemiol, 2011. **173**(7): p. 761-7.

6. Wong, J.J., et al., *Epidemiology of pediatric acute respiratory distress syndrome in singapore: risk factors and predictive respiratory indices for mortality.* Front Pediatr, 2014. **2**: p. 78.

7. Yehya, N., S. Servaes, and N.J. Thomas, *Characterizing degree of lung injury in pediatric acute respiratory distress syndrome.* Crit Care Med, 2015. **43**(5): p. 937-46.

8. Yehya, N. and N.J. Thomas, *Disassociating Lung Mechanics and Oxygenation in Pediatric Acute Respiratory Distress Syndrome.* Crit Care Med, 2017. **45**(7): p. 1232-1239.

9. Bodnar, L.M., et al., *Marginal structural models for analyzing causal effects of time-dependent treatments: an application in perinatal epidemiology.* Am J Epidemiol, 2004. **159**(10): p. 926-34.
